# Osteocyte Vegf-a contributes to myeloma-associated angiogenesis and is regulated by Fgf23

**DOI:** 10.1038/s41598-020-74352-x

**Published:** 2020-10-14

**Authors:** Patrick L. Mulcrone, Shanique K. E. Edwards, Daniela N. Petrusca, Laura S. Haneline, Jesús Delgado-Calle, G. David Roodman

**Affiliations:** 1 Department of Microbiology and Immunology, Indiana University School of Medicine, Indianapolis, IN USA; 2 Department of Medicine, Division of Hematology/Oncology, Indiana University School of Medicine, Joseph E. Walther Hall, Room C312, 980 W. Walnut St, Indianapolis, IN 46202 USA; 3 Department of Anatomy, Indiana University School of Medicine, Indianapolis, IN USA; 4 Indiana Center for Musculoskeletal Health, Indiana University School of Medicine, Indianapolis, IN USA; 5 Department of Pediatrics, Indiana University School of Medicine, Indianapolis, IN USA; 6 Roudebush VA Medical Center, Indianapolis, IN USA

**Keywords:** Myeloma, Tumour angiogenesis, Bone

## Abstract

Multiple Myeloma (MM) induces bone destruction, decreases bone formation, and increases marrow angiogenesis in patients. We reported that osteocytes (Ocys) directly interact with MM cells to increase tumor growth and expression of Ocy-derived factors that promote bone resorption and suppress bone formation. However, the contribution of Ocys to enhanced marrow vascularization in MM is unclear. Since the MM microenvironment is hypoxic, we assessed if hypoxia and/or interactions with MM cells increases pro-angiogenic signaling in Ocys. Hypoxia and/or co-culture with MM cells significantly increased Vegf-a expression in MLOA5-Ocys, and conditioned media (CM) from MLOA5s or MM-MLOA5 co-cultured in hypoxia, significantly increased endothelial tube length compared to normoxic CM. Further, Vegf-a knockdown in MLOA5s or primary Ocys co-cultured with MM cells or neutralizing Vegf-a in MM-Ocy co-culture CM completely blocked the increased endothelial activity. Importantly, Vegf-a-expressing Ocy numbers were significantly increased in MM-injected mouse bones, positively correlating with tumor vessel area. Finally, we demonstrate that direct contact with MM cells increases Ocy Fgf23, which enhanced Vegf-a expression in Ocys. Fgf23 deletion in Ocys blocked these changes. These results suggest hypoxia and MM cells induce a pro-angiogenic phenotype in Ocys via Fgf23 and Vegf-a signaling, which can promote MM-induced marrow vascularization.

## Introduction

Multiple myeloma (MM) is the 2nd most common hematological malignancy, with an estimated 32,000 new cases in 2020^1^. MM is characterized by the uncontrolled growth of malignant plasma cells in the bone marrow (BM). As MM develops, patients present with increased angiogenesis in the BM microenvironment, which favors myeloma cell infiltration and supports myeloma cell growth and survival. Further, > 80% of patients develop MM bone disease (MMBD) that results in severe bone-destructive lesions and markedly decreased bone formation. Thus, patients with MMBD are at an increased risk for a myriad of skeletal-related events, including pathologic fractures and severe bone pain that significantly affect their quality of life and survival^2–5^.

Interactions between MM and cells in the bone microenvironment play key roles in the development of MMBD through increased pro-survival gene expression in MM cells, elevated osteoclast activity, and decreased bone formation^6–13^. Recently, osteocytes (Ocy), the most abundant cell type in skeletal tissue, have been recognized as key contributors to MM cell growth and MMBD. Ocys communicate with MM cells via bidirectional Notch signaling that enhances the growth and survival of MM cells in bone both directly and indirectly^10,14,15^. Moreover, MM cells increase Ocy apoptosis, which promotes osteoclast formation and activity, and suppresses osteoblast differentiation via disruption of the OPG-RANKL axis and upregulation of sclerostin^11^.

Ocys perform essential functions in bone homeostasis and are unique reservoirs of proteins that regulate bone biology^14^. Our group and others showed that certain Ocy-specific proteins contribute to the clinical manifestations of MMBD^10,12^. Blocking the Ocy-specific protein Sclerostin prevents the bone destruction observed in MMBD with no effect on tumor burden^12,16^. Interestingly, circulating levels of the Ocy hormone Fibroblast Growth Factor 23 (Fgf23) are higher in MM patient sera compared to control patient sera. Fgf23 has been reported to increase levels of pro-oncogenic factors in MM^17^. Moreover, emerging evidence has shown that Ocys are pro-angiogenic and contribute to processes like fracture healing, yet their role in the elevated vascularization associated with MM development is unknown^12,18^.

Angiogenesis, the process of blood vessel development from the existing vasculature, is necessary for the growth of solid and hematological malignancies, and is an essential step in the metastatic cascade^3, 6,9^. The bone marrow microenvironment of MM patients is essential for MM progression and facilitates angiogenesis in part because it is extremely hypoxic^4,19^. Activated stromal cells in the MM microenvironment synthesize pro-oncogenic and pro-angiogenic factors that promote expansion and survival of MM cells, resulting in lower oxygen tension in the marrow and activation of angiogenic signaling pathways^4,6,7,19^. Data from MM patient samples reveal that marrow vascular density is increased in active MM patients compared to early stage or asymptomatic MM patients^3,6,7^. Further, elevated levels of marrow angiogenesis correlate with decreased MM patient survival, suggesting that development of MMBD and changes in vascularity in the bone microenvironment are linked^3^ While it is unknown whether osteocytes contribute to the observed increases in marrow vascular density in MM patients, it has been reported that hypoxia can increase levels of osteocyte-specific proteins Dmp1 and Mepe ex vivo^20^. Moreover, genetic deletion of osteocytic *Phd2*, a negative regulator of hypoxic signaling, in C57/Bl6 mice caused increased blood vessel formation in bone, suggesting Ocys are affected by alterations in oxygen tension and can induce vascular changes in the skeleton^21^. However, it has yet to be explored if osteocytes produce pro-angiogenic factors and promote angiogenesis in the context of MM.

Therefore, our objectives for this study were to determine if hypoxia and the presence of MM induce a pro-angiogenic phenotype in Ocys, to assess the angiogenic nature of Ocys in multiple myeloma-bearing bones, and to elucidate what osteocytic factors and signaling pathways potentially contribute to the enhanced angiogenesis associated with MM.

## Results

### Hypoxia increases pro-angiogenic gene expression in Ocys

**Figure 1:**
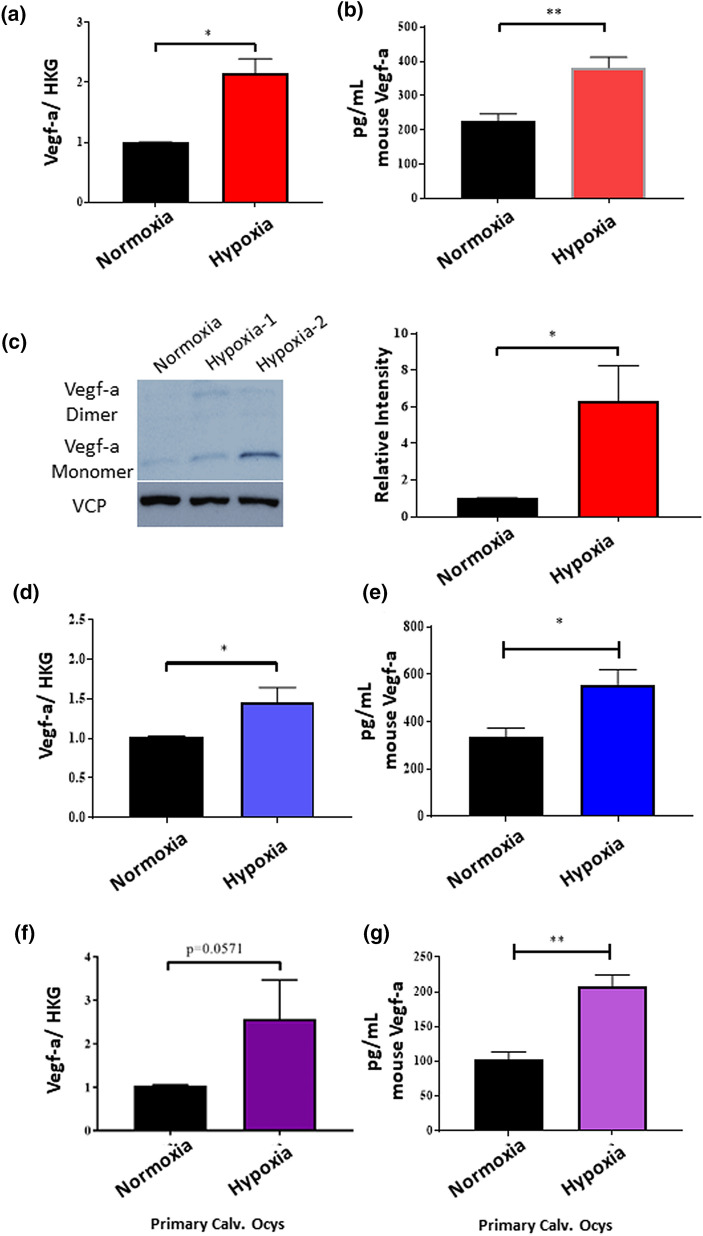
Hypoxia Increases Vegf-a Levels in MLOA5s, MLOY4s, and Primary Calvarial Osteocytes. (**a**) Confirmation of gene expression changes for Vascular Endothelial Growth Factor A (*Vegf-a)* in MLOA5s. N = 3–4. (**b**) Increased Vegf-a is secreted into media of hypoxic MLOA5s. N = 6. (**c**) Representative western blot images of normoxic (Lane 1) and hypoxic (Lanes 2, 3 biological replicates) MLOA5 osteocytes run on the same gel cropped for publication purposes. N = 4 for Western blot analysis. Full-length gel is provided as Supplemental Fig. S8. (**d**) qPCR for *Vegf-a* in MLOY4s cultured in Normoxia or Hypoxia (1%) for 24 h. (**e**) ELISA for Vegf-a from conditioned media of MLOY4s. N = 3–4 for both studies. (**f**) qPCR for *Vegf-a* in Primary Calvarial Osteocytes cultured in Normoxia or Hypoxia (1%) for 24 h. (**g**) ELISA for Vegf-a from conditioned media of Primary Calvarial Osteocytes. N = 4 for both studies. (* = *p* < 0.05, ** = *p* < 0.01).

A known feature of tumor microenvironments that promotes angiogenesis is hypoxia^7,13,19,22^. Therefore, we first tested in vitro if hypoxic culture conditions increased angiogenic gene expression in murine osteocytic cells. We cultured MLOA5 murine Ocy-like cells in normoxia (21%O_2_) or hypoxia (1%O_2_) for 24 h then assessed angiogenic gene expression profile of Ocys. We found that the expression of several pro-angiogenic genes was increased in the hypoxic cells (including Angpt2, Efnb2, and Igf; Supplemental Fig. S1). *Vegf-a*, a known pro-angiogenic and pro-oncogenic growth factor, was upregulated 2.67 fold in the hypoxic MLOA5s compared to normoxic cells. The increase in *Vegf-a* expression found in hypoxic MLOA5s was validated by qPCR at the mRNA level, and by Western blot and ELISA at the protein level (Fig. 1a–c). Moreover, we found similar results in a separate murine Ocy cell line, MLOY4s, and in primary Ocys cultured from 12-week old mouse calvariae (Fig. 1d–g). These in vitro results show that Ocys upregulate Vegf-a in response to hypoxia.

### Vegf-a secreted by Ocys promotes vessel formation and endothelial migration in vitro

**Figure 2:**
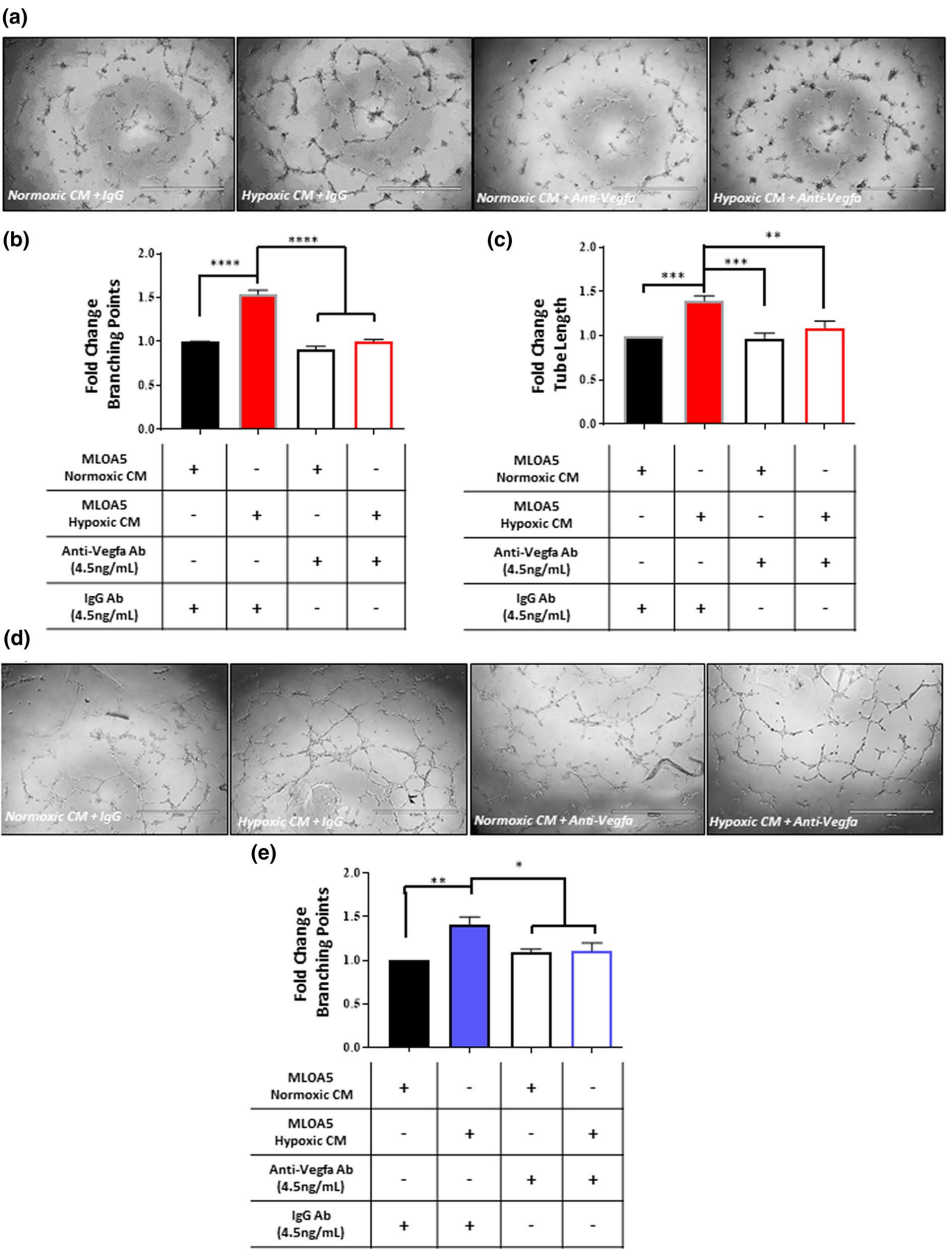
Addition of anti-Vegf-a Antibody to Hypoxic Osteocyte Conditioned Media Reduces Tube Formation in vitro. (**a**) Representative 4 × images of 5000 HUVECs. Hypoxic conditioned media (CM) group exhibits greater vascular complexity compared to other 3 groups. (**b**) Branching point analysis and (**c**) Tube length calculations are increased in Hypoxic CM treated HUVECs. N = 5. (**d**) and (**e**) A similar pattern is observed using primary Endothelial Colony Formation Cells (ECFCs). (* = *p* < 0.05, ** = *p* < 0.01, *** = *p* < 0.001, **** = *p* < 0.0001 by ANOVA).

To test in vitro the functionality of the secreted Vegf-a produced by Ocys, we harvested conditioned media (CM) from normoxic and hypoxic MLOA5s and treated HUVEC cells in the standard tube formation assay grown on basement membrane extracts. We observed an increase in branching points and tube length in HUVECs treated with the MLOA5-hypoxic CM (Supplemental Fig. S2). Next, we tested the specific contribution of Ocy-derived Vegf-a in promoting the HUVEC tube formation using a neutralizing antibody specific for murine Vegf-a. The increase in HUVEC network formation induced by hypoxic Ocy-CM was abrogated in the presence of the Vegf-a neutralizing antibody. These results support that Ocys are pro-angiogenic cells, and osteocytic Vegf-a is responsible for the vascular changes observed in HUVEC tube assays (Fig. 2a–c). Further, neutralizing osteocytic Vegf-a activity reduced HUVEC migration induced by hypoxic MLOA5 CM (Supplemental Fig. 3). Importantly, similar results also were seen using primary human ECFCs (Fig. 2d,e).

### Direct contact between Ocys and multiple myeloma increases osteocytic Vegf-a secretion

**Figure 3:**
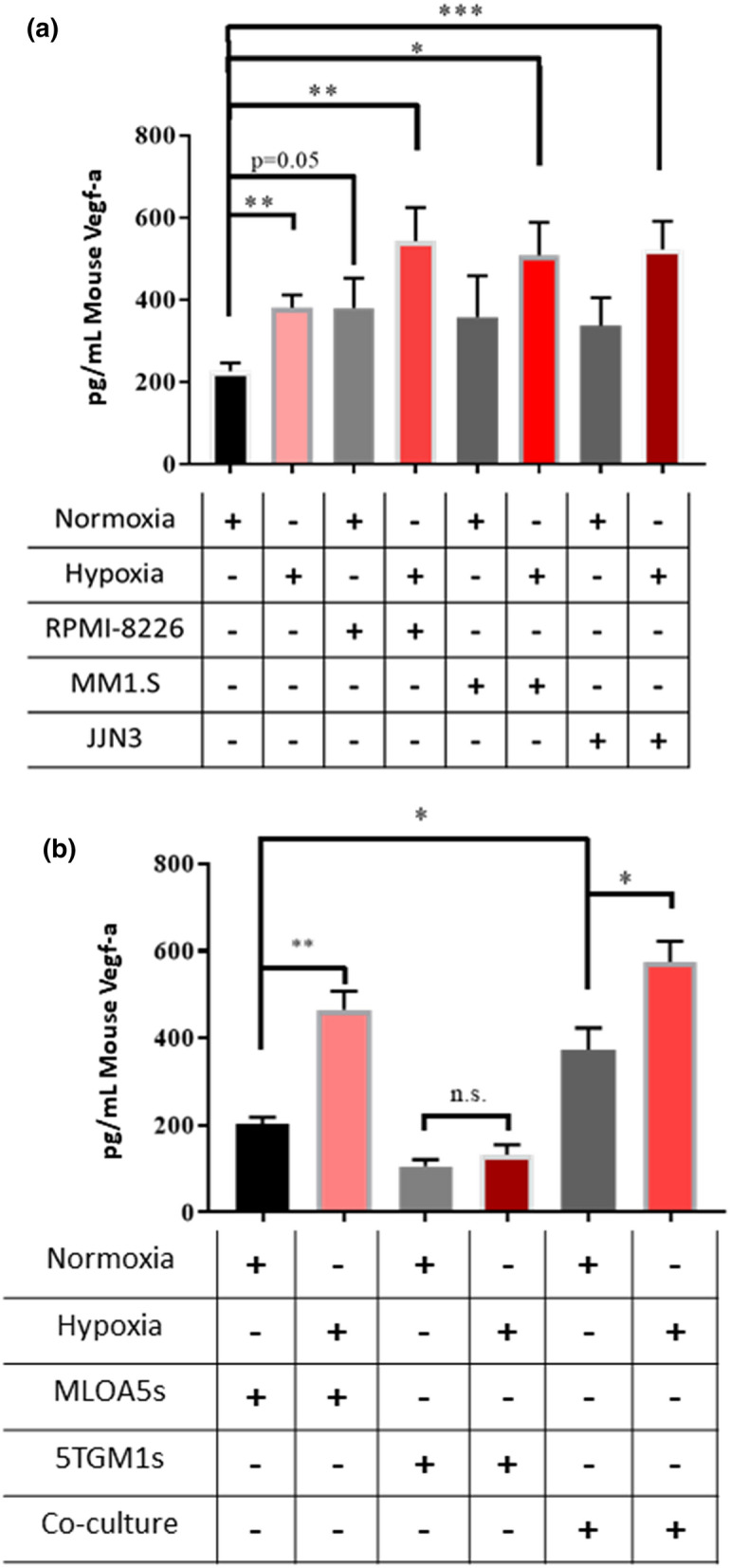
Co-culture of Human MM in Either Normoxia or Hypoxia Increases Vegf-a Secretion by MLOA5 Osteocytes after 24 h. (**a**) Murine Vegf-a levels detected by ELISA from varying conditioned media. MM cell lines used: RPMIs, MM1.S, JJN3s. N = 4–6. (**b**) A similar pattern is seen with 5TGM1 murine MM co-cultures. N = 3–4, (* = *p* < 0.05, ** = *p* < 0.01, *** = *p* < 0.001).

Many groups have demonstrated that the growth and progression of several cancers in bone are dependent on direct interactions between cancer cells and the cells of the bone-BM microenvironment^11,12,19,23^. Our group has shown that direct contact between MM and Ocys occurs in MM-involved marrow and induces signaling that promotes osteoclast activity and decreases osteoblast differentiation; both are clinical aspects of MMBD^10^. Therefore, we determined if direct interaction between Ocys and MM cells also induced a pro-angiogenic phenotype in the MM niche. As shown previously, we observed a significant increase in secreted Vegf-a in monocultures of MLOA5s in hypoxia compared to normoxia (Fig. 3a). Co-culture of MLOA5s with human MM cells (RPMI-8226, MM1.S, or JJN3) for 24hrs also increased murine Vegf-a by 67%, 58%, and 49% respectively, compared to MLOA5 cells cultured alone and in normoxic conditions (Fig. 3a). When these co-cultures were performed in hypoxia, the amount of secreted murine Vegf-a more than doubled in each system, demonstrating that low oxygen and direct interaction with MM cells, two components of the MMBD microenvironment, stimulate Vegf-a secretion by Ocys (Fig. 3a).

To examine if MM cells were also producing Vegf-a in response to hypoxia and in co-cultures with Ocys, we performed co-culture experiments with the murine MM cell line 5TGM1. Unlike the MLOA5s cells, there was no difference in 5TGM1-derived Vegf-a production between normoxic and hypoxic conditions. Of note, the amount of Vegf-a produced by the 5TGM1s was half of the amount secreted by the MLOA5 Ocys (Fig. 3b). Co-culture of MLOA5 and 5TGM1 cells exhibited a similar pattern and Vegf-a production as the co-cultures with human MM cells in Fig. 3a. These results show that hypoxia induces a pro-angiogenic phenotype in Ocys via elevated Vegf-a secretion, but not in murine MM cells, and contact with MM cells further increases osteocytic Vegf-a secretion.

### Vegf-a-expressing Ocys are significantly increased in bones of mice harboring multiple myeloma cells

**Figure 4:**
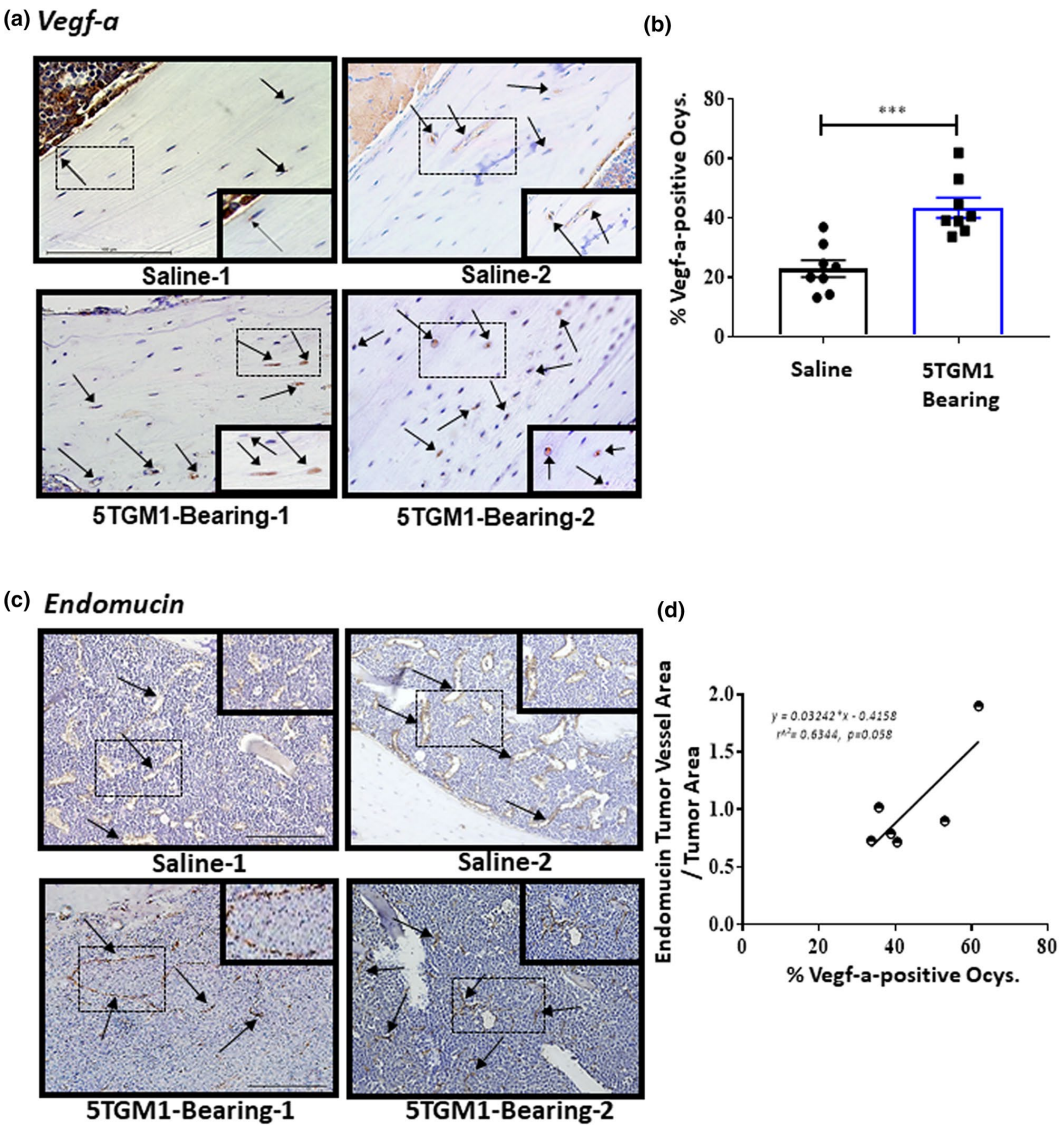
Mice Harboring MM Have Increased Percentage of Vegf-a-positive Osteocytes, which Correlates with Tumor Vessel Area. (**a**) Representative 40 × images, from two separate mice in each group, of Vegf-a-stained murine tibiae injected intratibially with saline or 10^5^ 5TGM1 multiple myeloma (MM) cells. Dotted black box indicates zoomed in area of each section. Black arrows indicate Vegf-a-positive osteocytes. (**b**) Percentage of Vegfa-positive osteocytes 4 weeks after inoculation. N = 8 for each group. (*** = *p* < 0.001). (**c**) Representative 20 × images of tibiae from two separate mice in each group, stained with Endomucin. Dotted black box indicates zoomed in area of each section. Black arrows indicate Endomucin-positive blood vessels. (**d**) Correlation between percent Vegf-a-positive osteocytes and Endomucin-positive tumor vessel area in 5TGM1 MM-bearing mice. (N = 6, *p* = 0.058).

To determine if the in vitro observations are mirrored in vivo*,* we utilized an intratibial murine model of multiple myeloma that recapitulates the traits of human MMBD^10,13,16^. We analyzed sections of bones containing MM cells for Vegf-a protein expression and blood vessel density via immunohistochemistry (IHC). 5TGM1 tumor-bearing mice exhibited a twofold increase in Vegf-a positive Ocys compared to saline injected controls (Fig. 4a,b). We also found that the percentage of Vegf-a-expressing Ocys positively correlated with Endomucin (EMCN) positive tumor vessel area in our 5TGM1-bearing bones (Fig. 4c,d). Importantly, we also observed this increase in Vegf-positive Ocys via IHC in a second in vivo MM study in which human JJN3 cells were injected intratibially into Scid mice (Supplemental Fig. S4). These in vivo results are consistent with our in vitro observations and support the hypothesis that MM cells in bone stimulate Vegf-a production in Ocys.

### Neutralization of osteocytic Vegf-a diminishes their angiogenic phenotype in in vitro and ex vivo co-cultures with myeloma

To investigate whether inhibition of Vegf-a affects the pro-angiogenic phenotype of Ocys in the presence of MM, we used pharmacological and genetic approaches to manipulate Ocy Vegf-a. HUVECs treated with hypoxic CM from human MM and murine MLOA5 co-culture experiments developed a more complex vascular networks in vitro compared to normoxic CM. Treatment with a murine Vegf-a neutralizing antibody fully blocked this effect, demonstrating that osteocytic Vegf-a from the co-cultures is responsible for the vascular changes in vitro (Supplemental Fig. S5).

**Figure 5:**
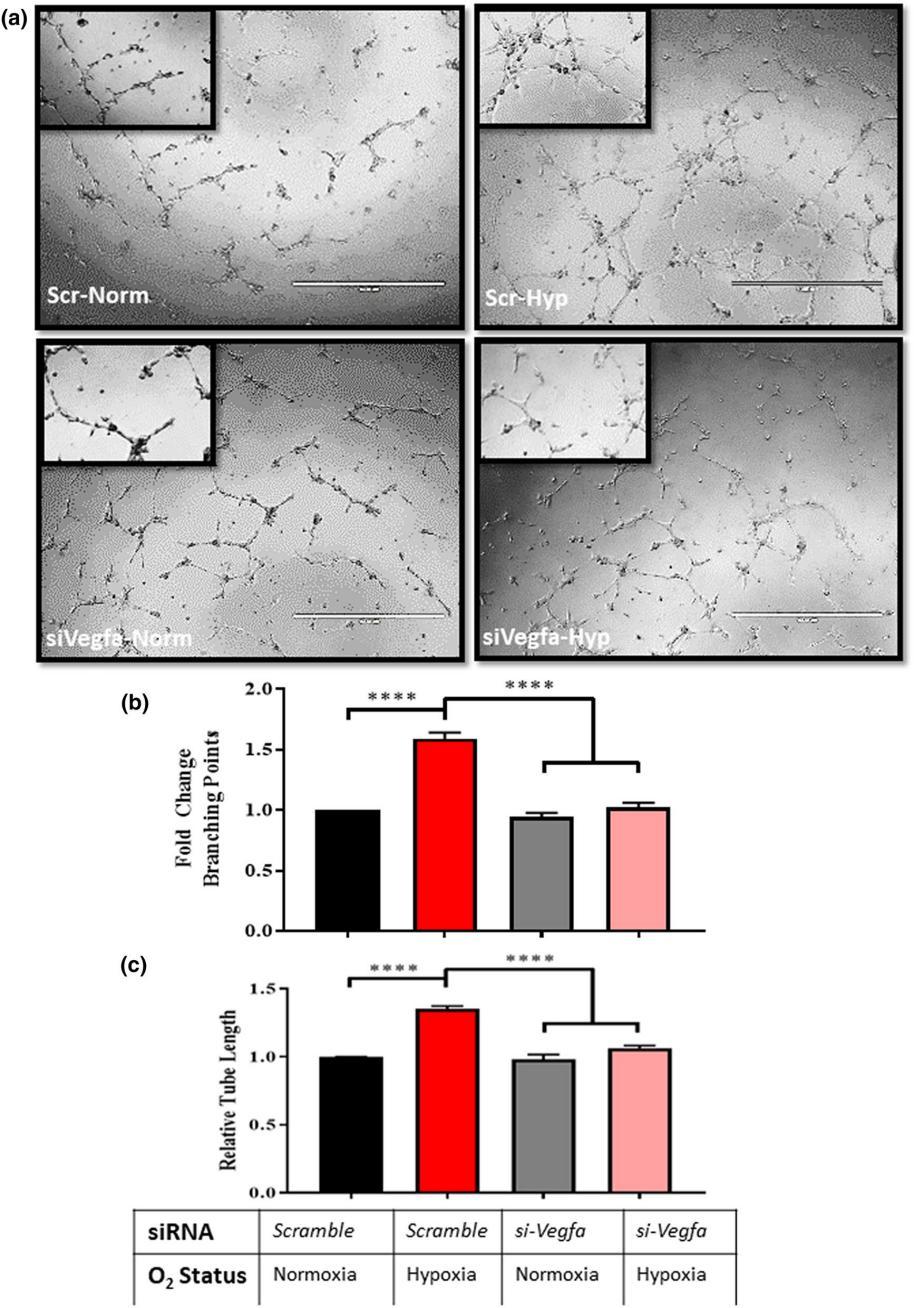
Knockdown of *Vegf-a* in Hypoxic Osteocytes Blunts HUVEC Vessel Formation in vitro. (**a**) Representative 4 × images with zoomed in insert of HUVEC networks. (**b**) Branching point and C) Tube Length analyses reveal increased vascular complexity in Scr-Hypoxia group compared to other 3 groups. N = 3 (**** = *p* < 0.0001 by ANOVA).

**Figure 6:**
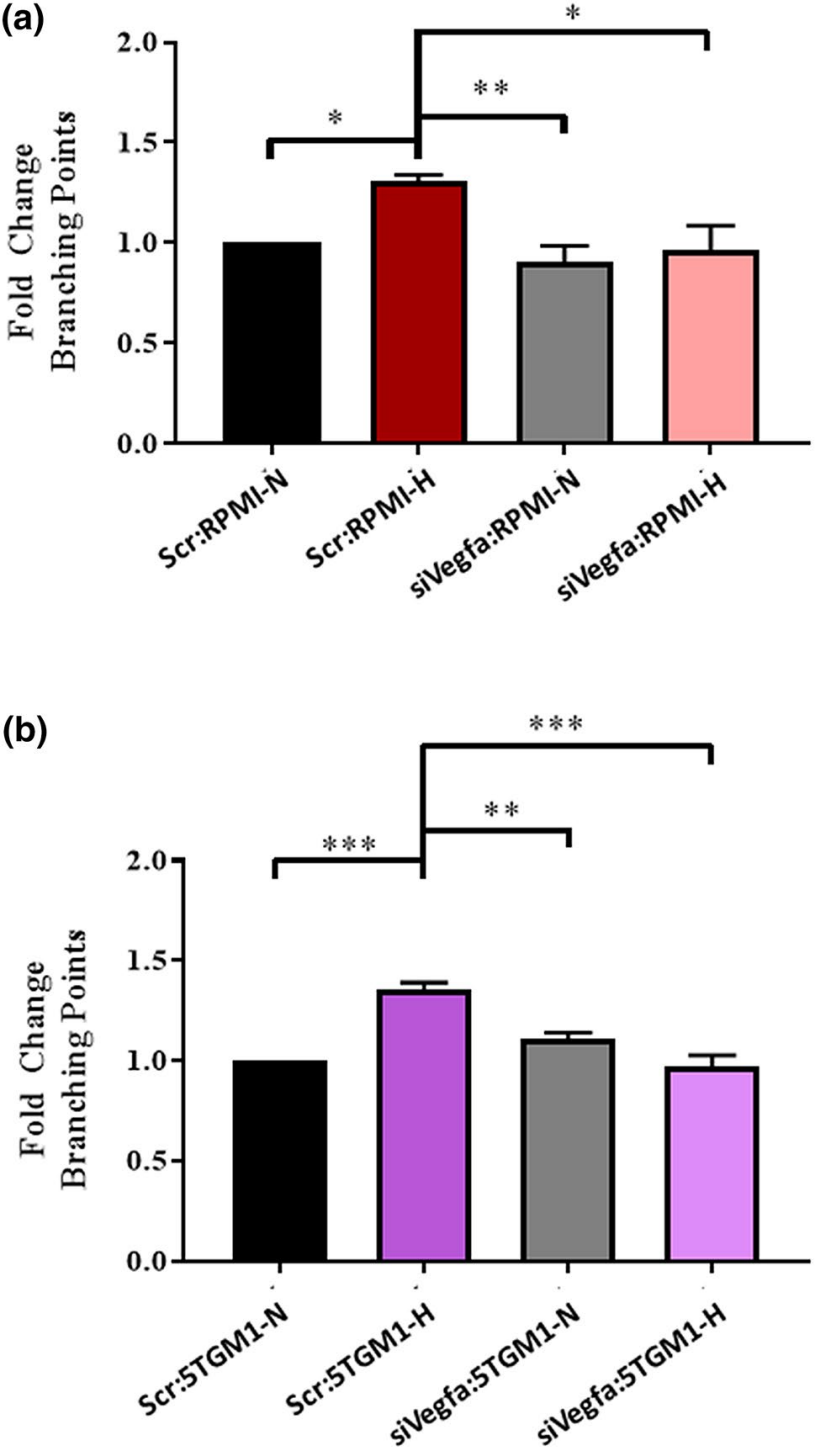
Conditioned Media from MM:siVegfa-MLOA5 Co-Cultures Does Not Induce HUVEC Vessel Formation: (**a**) Fold Change in branching point analysis of HUVECs treated with RPMI:MLOA5 co-culture CM at 8 h. N = 4. (**b**) Fold Change in branching point analysis of HUVECs treated with 5TGM1:MLOA5 co-culture CM at 8 h. N = 3, (* = *p* < 0.05, ** = *p* < 0.01, *** = *p* < 0.001 by ANOVA).

**Figure 7:**
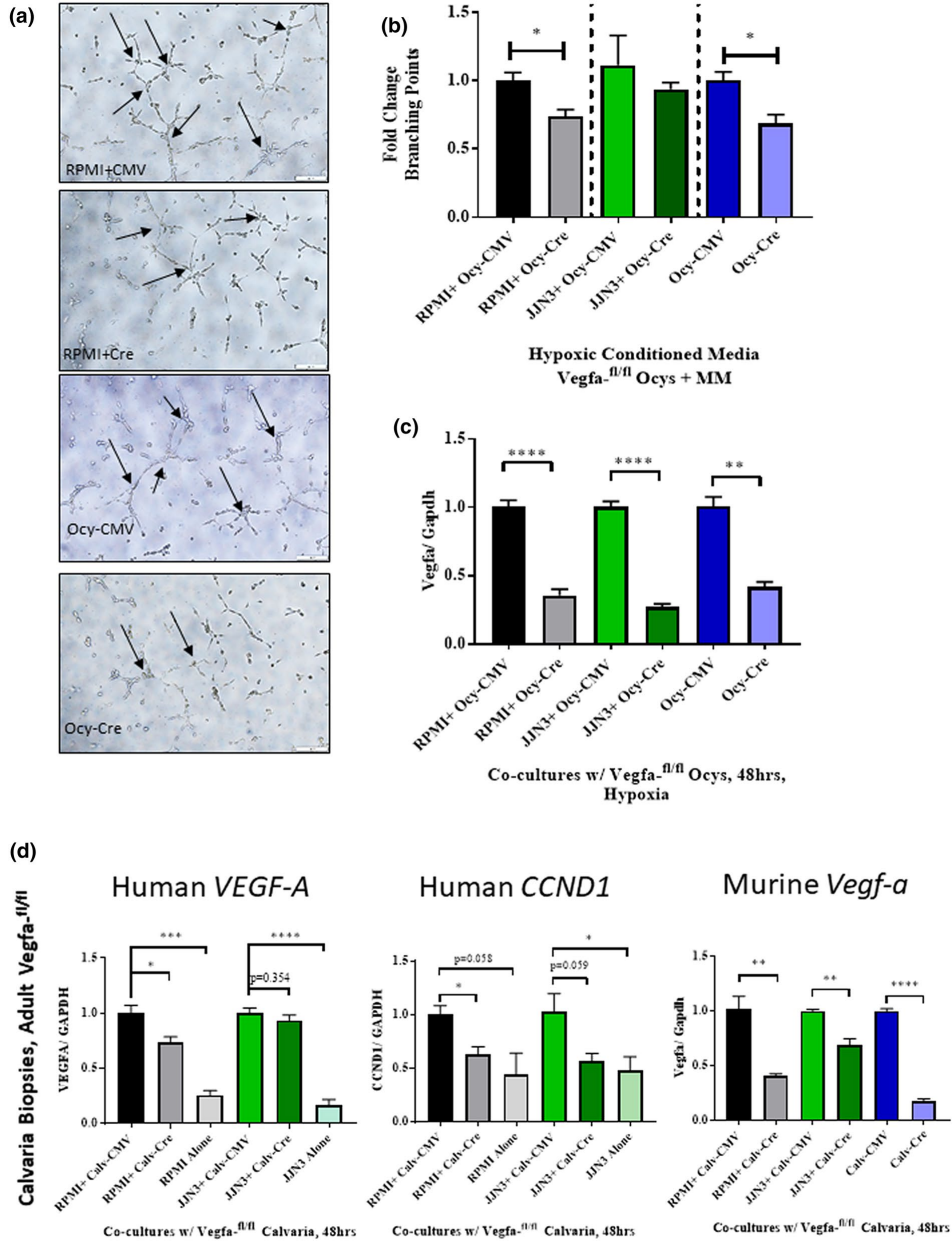
Knockdown of Osteocytic *Vegf-a* Reduces Tube Formation and MM Expression of VEGF-A and CCND1 in EVOCA:MM Co-Culture Model: (**a**) Representative 10 × images of HUVECs treated with hypoxic CM from designated co-culture groups. (**b**) Quantification of branching points. N = 3. (**c**) Murine *Vegf-a* expression shows multiplicity of infection (MOI) of 120 reduces murine Vegf-a in primary osteocytes N = 3. (**d**) Expression of human *VEGF-A* and *CCND1* do not increase in RPMIs and JJN3s co-cultured with Cre-treated bone nor in MM mono-culture controls. Murine *Vegf-a* levels are decreased with Cre transduction. N = 3–6. (* = *p* < 0.05, ** = *p* < 0.01, *** = *p* < 0.001, **** = *p* < 0.0001).

Moreover, using siRNAs targeting Vegf-a, we achieved a 55% knockdown of Vegf-a expression at the mRNA level in Ocys and tested the ability of these cells to promote angiogenesis (Supplemental Fig. S6a-b). MLOA5 viability was unaffected by the siRNA, and expression of *Vegf-a* was significantly decreased in our siVegfa groups, regardless of oxygen tension (Supplemental Fig. S6c-d). As expected, we observed increased HUVEC network formation when using CM from scr-Ocy-Hypoxia group compared to scr-Ocy-Normoxia. In contrast, these effects were fully prevented when CM from Ocys transfected with siVegfa was used, a result that mirrors our pharmacological studies (Fig. 5). Next, we investigated whether interactions with MM cells can compensate for the lack of osteocytic Vegf-a to induce tube formation in vitro. We observed increased branching points in the Scr-Hypoxia groups compared to Scr-Normoxia. Regardless of oxygen tension, CM from siVegfa-MLOA5:MM co-cultures did not promote HUVEC tube formation (Fig. 6, Supplemental Fig. S7). Further, we collected CM from co-cultures established with primary murine Ocys isolated from adult Vegfa-^*flox/flox*^ mouse bones treated with or without Adeno-Cre-eGFP (Ad-Cre) virus and human MM cells. Treatment with Adeno-Cre decreased Vegf-a expression in Ocys by 60–75% (Fig. 7c). CM from Ad-Cre treated primary Ocys isolated from adult Vegfa-^*flox/flox*^ mice induced significantly less tube formation in HUVEC cultures compared to Ad-CMV treated cells. A significant reduction in tube formation was also observed using CM from 48 h co-cultures between Vegfa-^*flox/flox*^ Ocys and RPMI cells, and a slight reduction was detected in the Vegfa-^*flox/flox*^ Ocy:JJN3 co-culture (Fig. 7a,b). These results demonstrate that osteocytic Vegf-a induced by hypoxia is responsible for the observed pro-angiogenic effect, and MM cells are unable to compensate for the absence of osteocytic Vegf-a in hypoxia in vitro.

To address our question using a system that resembles the complexity of the interactions in the MM niche, we utilized an ex vivo bone organ culture (EVOCA) model^10,17^. Bone biopsies of adult Vegfa-^*flox/flox*^ mouse calvaria were co-cultured with 50,000 human MM cells (EVOCA) for 48 h and transduced with Ad-Cre to knockdown Vegf-a prior to the addition of MM cells. Control biopsies were transduced with Adeno-CMV-eGFP (Ad-CMV) and had intact Vegf-a. We demonstrated that myeloma *VEGF-A* levels did not increase in the Ad-Cre-treated EVOCA co-cultures compared to the control Ad-CMV-treated group, using species-specific primers, a result that supports our ELISA data. Interestingly, we observed a decrease in human *CCND1*, a known driver of MM pathogenesis in both RPMIs and JJN3s in the Ad-Cre treated EVOCAs (Fig. 7d)^24^. These results further suggest a key role for osteocytic Vegf-a in supporting MM growth ex vivo and promoting vascularization in vitro.

### Fgf23 promotes osteocytic Vegf-a production

**Figure 8:**
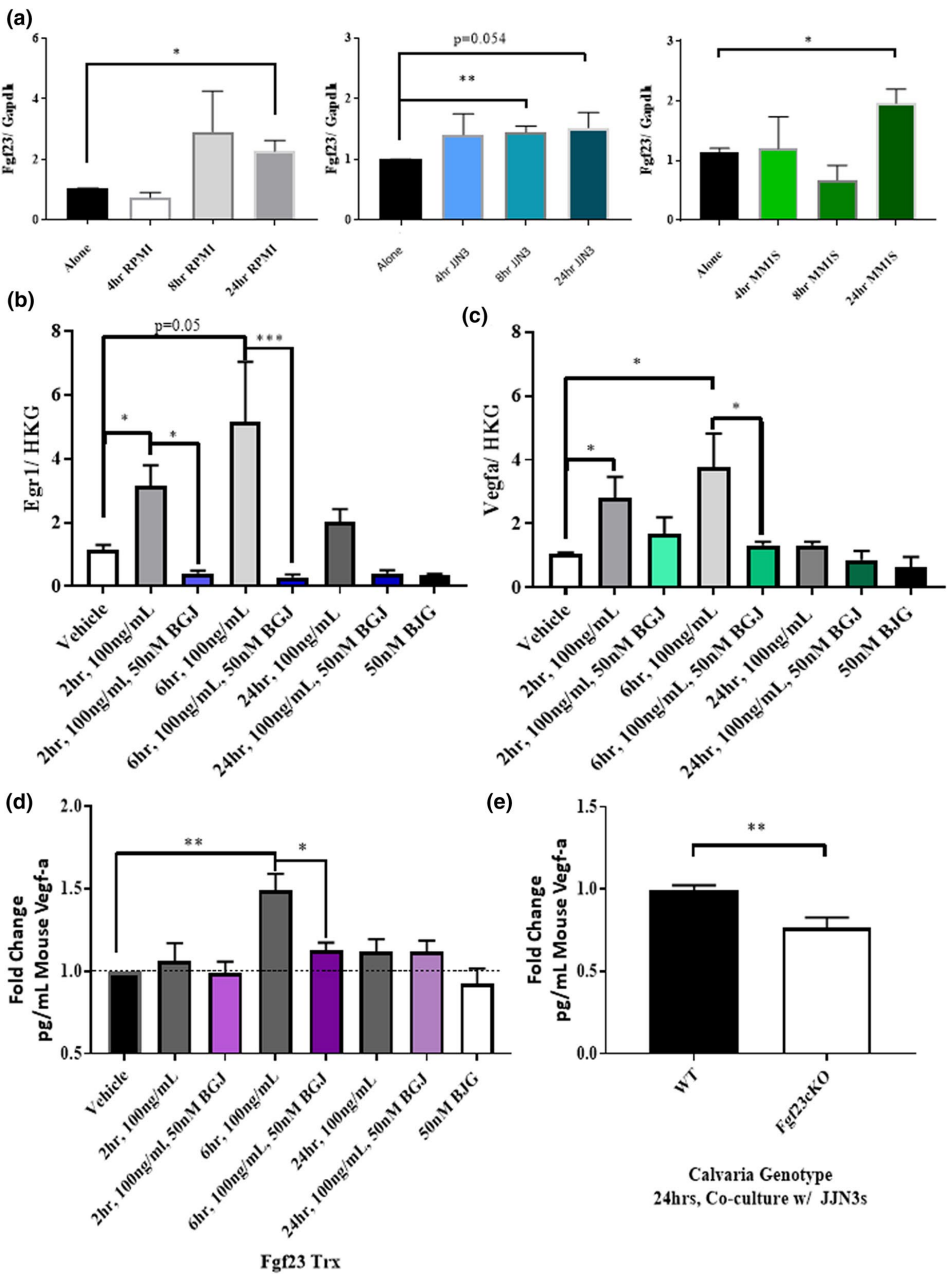
Fgf23 Enhances Vegf-a Expression and Secretion by Osteocytes. (**a**) RPMI (Gray), JJN3 (Blue), & MM1.S (Green) myeloma cells induce Fgf23 expression in MLOA5s. N = 3–6. (**b**) *Egr1* expression in MLOA5s increases with 100 ng/mL of Fgf23 treatment and is reduced with 50 nM BGJ398 treatment. (**c**) *Vegf-a* expression in MLOA5s follows the same pattern. N = 3–4 for B & C. Vehicle treatment time is 24 h. (**d**) Vegf-a ELISA shows Fgf23 Induces Secretion of Vegf-a in MLOA5 Osteocytes. This is reduced with 50 nM BGJ398 at 6 h. N = 6. (**e**) Osteocyte-enriched calvaria from Fgf23cKO mice secrete less Vegf-a in the presence of JJN3s via ELISA. N = 9. (* = *p* < 0.05, ** = *p* < 0.01, *** = *p* < 0.001).

Ocys are the main producers of Fgf23, an essential protein for phosphate homeostasis and bone health^14,25–27^. Previous work demonstrated that elevated Fgf23 is detectable in the sera of MM patients and is linked to development of MMBD in ex vivo models^17^. Consistent with these results, we found in vitro that direct interaction between several human myeloma cell lines and MLOA5 murine Ocys caused increased expression of murine *Fgf23* (Fig. 8a).

Early Growth Response 1 (Egr1), which is a downstream target of Fgf23, can bind the Vegf-a promoter and transcribe *Vegf-a* in several cell types^28,29^. Thus, we chose to determine if the Fgf23–Egr1 axis regulated osteocytic Vegf-a upregulation. Treatment with Fgf23 increased *Egr1* expression in MLOA5s after 2, 6, and 24 h treatments; these increases were blocked with the addition of BGJ398, a pan-FGF receptor inhibitor (Fig. 8b). Treatment with Fgf23 also induced a significant increase in *Vegf-a* expression that was blunted by BGJ398 (Fig. 8c). This inhibitor also blocked Fgf23-induced Vegf-a secretion by MLOA5s (Fig. 8d). To assess how the loss of Fgf23 affects Vegf-a levels in MMBD, we established EVOCA systems by co-culturing human JJN3 MM cells with calvarial biopsies isolated from WT mice or mice that lack Fgf23 specifically in osteocytes (Fgf23cKO mice)^30^. After 24 h, we observed a significant reduction in murine Vegf-a production in the JJN3:Fgf23cKO EVOCA compared to JJN3:WT controls by ELISA (Fig. 8e). These results suggest that autocrine Fgf23 can induce *Vegf-a* and *Egr1* expression in Ocys and contributes to Vegf-a production in the presence of MM.

## Discussion

The development of multiple myeloma bone disease (MMBD) is dependent on interactions between the MM cells and the many cell types in the bone microenvironment^7,19^. MMBD is characterized by increased osteoclast activity, dysfunctional osteoblast activity, and elevated bone marrow angiogenesis. Ocys, the most abundant cell type in bone tissue, have been shown to support MMBD directly by promoting MM growth and osteoclast formation, while blocking osteoblast activity^10–15^. Here, we provide new evidence that Ocys exposed to hypoxia and in direct contact with MM cells produce the pro-angiogenic growth factor *Vegf-a* and promote vessel formation in vitro and ex vivo. Furthermore, Fgf23 regulates the production of osteocytic Vegf-a in an autocrine manner. In two mouse models of MMBD, we observed an elevated number of Vegfa-positive Ocys compared to their saline-injected counterparts, which positively correlates with MM vascular density. Our in vitro, ex vivo, and in vivo results demonstrate new evidence that the hypoxic microenvironment in the MM niche reprograms Ocy towards a more pro-angiogenic phenotype through elevated production of Vegf-a that is promoted by Fgf23. Mechanistic studies using pharmacological and genetic approaches demonstrate that Ocy-derived Vegf-a is sufficient to stimulate the migration of endothelial cells and the formation of a vascular network, fundamental steps for angiogenesis. Interestingly, our studies show that Ocys produce more Vegf-a than MM cells in hypoxia. Together, these findings support the notion that Ocys may be an important source of Vegf-a in the MM niche and possibly key contributors to the increased angiogenesis in MM via a novel Fgf23-Vegfa signaling axis.

Several cytokines and pro-angiogenic growth factors have been identified and implicated in tumor progression and angiogenesis^6,7,19,22^. However, VEGF-A is the prime growth factor responsible for physiological and pathological angiogenesis^31,32^. Angiogenesis is a hallmark of cancer, and it requires coordinated crosstalk between several stromal cell types and the cancer cells^33^. The tumor microenvironment associated with MM is very hypoxic and facilitates angiogenesis to increase MM growth^6,7^. Ocys are sensitive to changes in oxygen tension and produce pro-angiogenic factors during mechanical loading and shear stress, and in the presence of pro-inflammatory cytokines^21,34–38^. Intriguingly, Cheung et al. report that Ocy apoptosis elevated Vegf-a secretion in viable Ocys and promoted in vitro tube formation^18^. Although we do not explore the role of osteocyte apoptosis in the current study, we and others have shown that MM cells increased osteocyte apoptosis as the disease progresses in both mouse models and patient samples^10,11^; this might be a complimentary mechanism to our current data of how Ocys induce MM angiogenesis. In addition, Stegen et al. show that genetic deletion of *Phd2*, a negative regulator of Hif1α, specifically in Ocys caused cortical bone growth and elevated cortical vascular density in vivo^21^. This data demonstrate how activation of hypoxic signaling pathways induces pro-angiogenic changes in Ocys and supports the ELISA data we present in this manuscript. We observed that MM cells in vitro secrete less Vegf-a compared to Ocys in both normoxia and hypoxia. Our data also showed that hypoxia did not increase Vegf-a production by MM cells, suggesting that the low oxygen tension of the MMBD microenvironment has a more profound effect on osteocytic Vegf-a production than on MM Vegf-a^37^.

Once tumors develop a vascular network, they grow exponentially, are more difficult to treat, and are more likely to spread to other areas of the body. Clinical reports of MM demonstrate that neovascularization is higher in patients with diffuse MM compared to early stage patients and healthy patients^6,7,38,39^. Moreover, interactions between myeloma cells and endothelial cells lead to disease progression, partially regulated by myeloma-associated endothelial Notch signaling. These tumor-endothelial cell interactions are also implicated in drug resistance in MM, as well as lung cancer and pancreatic islet cell carcinoma^40–42^. Our data demonstrate that Ocys produce Vegf-a in response to hypoxia and direct contact with MM, thus altering the vascular landscape of MM niche and possibly contributing a potential mechanism of drug resistance in MM that should be explored further.

Further studies are warranted to determine the specific contribution of Ocy-derived Vegf-a versus MM-derived Vegf-a to the increased angiogenesis in bones bearing MM cells. Translational studies report that MM cells are able to produce Vegf-a and that blocking Vegf-a signaling using a VEGFR2 neutralizing antibody can improve survival in mouse models of MM^40,43,44^. However, because we observed that MM cells in vitro secrete less Vegf-a compared to Ocys (Fig. 3), the effects of targeting VEGFR2 could be due to blocking stromal Vegf-a signaling more so than solely MM Vegf-a. Several FDA-approved drugs exist that target distinct aspects of angiogenesis, and bevacizumab has been used to treat MM patients usually in combination with chemotherapy or bortezomib, with limited success^45–47^. Nevertheless, several Vegf-targeting therapeutics are being explored for MM^48–50^. In addition, targeted delivery of anti-tumor and anti-vascular therapies using bone and vascular-specific binding motifs are showing promising efficacy in translational studies. Indeed, bone-targeting bortezomib demonstrated improved efficacy in targeting MM as well as reduced side effects compared to conventional bortezomib^51–53^. As angiogenesis is a key step in MM progression and interactions between MM and ECs can confer drug resistance, a similar approach in designing and testing bone-targeting anti-angiogenic drugs may be beneficial for MM patients. This approach could minimize toxicity associated with systemic anti-angiogenic therapies, target stromal reservoirs of Vegf-a, such as that of Ocys, and potentially enhance the effects of current first-line drugs for MM such as bortezomib^48^.

There are several details surrounding the contribution of Ocys to MMBD vascularization that need to be elucidated. Ocys interact with multiple cells of the bone microenvironment. Therefore, other bone cells that support MM growth and angiogenesis, such as osteoclasts, osteoblasts, and tumor-associated ECs, could be affected by the angiogenic signaling of Ocys. Previous work from our lab and others showed that conditioned media from primary human osteoclasts alone or co-cultured with MM cells stimulated blood vessel formation in vitro and ex vivo^54,55^. Tumor-associated ECs have been reported to support VEGF-A signaling in both lung cancer and MM models^40,41,44^. Future in vivo MM studies using an inducible *Cre:flox* mouse model that causes osteocyte knockout (*Dmp1-Cre*) of Vegf-a or Fgf23 are needed to determine the relative contributions of this cell type to the elevated marrow angiogenesis observed in MMBD^21,27,32,56–58^. Additionally, osteoblasts were reported to promote breast cancer bone metastasis via Vegf-a-mediated changes in bone vasculature^23^. However, the relatively greater abundance of Ocys compared to these other bone cells and tumor-ECs in a tumor microenvironment, and their well-studied homeostatic nature imply that Ocys potentially may alter angiogenesis to a greater degree and through several mechanisms. Another consideration that may affect the pro-angiogenic phenotype of osteocytes is the 3D environment in which they reside. Osteocytes are present in a 3D mineralized matrix that is subject to mechanical loading and forces known to alter their biology and production of cytokines^18,35,36^. Mechanical loading could further enhance the pro-angiogenic response of osteocytes to hypoxia and interactions with MM cells shown in our studies^34^.

Notch signaling controls vascular development, bone physiology and vascular development in the marrow, and is aberrant in several cancers^56–60^. Guo and colleagues reported that Notch1 overexpression in MM cells increases Vegf-a production and tumor vascular density in vivo^61^. Moreover, our group has demonstrated that bidirectional Notch signaling occurs between Ocys and MM, causing bone destruction and supporting MM survival, suggesting that osteocytic Notch signaling could contribute to MMBD vascularization. An in vivo MM study in mice lacking Vegf-a strictly in Ocys could demonstrate how the absence of this Vegf-a pool affects MM growth and development, potentially through a Notch signaling mechanism that is beyond the scope of the current study. It is also known that tumor cells exhibit close interactions with endothelium via adhesion protein interactions^40,62^. It is unclear whether Ocys can alter the adhesion profile of either MM or endothelial cells to promote seeding and growth.

Perturbations in Fgf23 signaling are linked to multiple pathologies that involve bone, as low levels of Fgf23 characterize Hyperphosphatemia and soft tissue calcification disorders. High levels of Fgf23, on the other hand, are observed in patients with chronic kidney disease (CKD) and hypophosphatemic Ricketts, which lead to poor bone quality, osteomalacia, and disrupted skeletal homeostasis^27,30,63^. Our data reveal that Fgf23 directly regulates Vegf-a production in Ocys, suggesting a novel angiogenic role for Fgf23 not reported previously. Increased levels of Fgf23 are detected in the sera of MM patients, and the source of Fgf23 is the bone microenvironment, as several MM cell lines do not express Fgf23^17^. S. Feng and colleagues demonstrated that prostate cancer, another tumor that thrives in the bone microenvironment, respond to Fgf23 by increased growth and MAPK signaling^64^. This reinforces the importance of the stroma, and in conjunction with our results, the Ocys, in the progression of tumors that grow in bone such as MM. Fgf23-targeting drugs, like Burosumab, might be efficacious in treating MMBD and reducing marrow neo-vascularization associated with its development^65^. Although the in vivo data presented in this manuscript supports the hypothesis that osteocytes are pro-angiogenic in MM microenvironment, these models have limitations and do not recapitulate all aspects of the human pathophysiology of MM^10,13,16,17,22^. In future studies, other modes of MM inoculation can be used to investigate how MM homing and establishment are affected by pro-angiogenic signaling in Ocys. Follow-up studies will also explore the effects of patient-derived MM cells on inducing a pro-angiogenic phenotype in osteocytes, as well as immunohistochemical analyses of clinical samples from patients with MMBD.

In conclusion, our results demonstrate that Ocys are pro-angiogenic cells in response to hypoxia and the MM microenvironment, and reveal that Fgf23 can induce this phenotype though the activation of an Egr1-Vegf-a signaling axis. This pool of Vegf-a induces blood vessel formation in vitro and ex vivo, as MM Vegf-a is not affected by hypoxia or contact with Ocys. Also, the percentage of Vegf-a-expressing osteocytes correlates with MM tumor vascularity in vivo, and Fgf23, which is made by Ocys, increases Vegf-a production via autocrine signaling and Egr1 activity. As there is no cure for MMBD, our work suggests improving anti-angiogenic drugs and targeting osteocytic Vegf-a and/or Fgf23 could be an effective therapeutic approach for MM patients.

## Methods

### Cell culture of cell lines and primary human endothelial colony formation cells (ECFCs)

MLOA5 and MLOY4 osteocyte-like cells (generously provided by Dr. Jesús Delgado-Calle and Dr. Lynda Bonewald, Indiana University School of Medicine [IUSM]) were cultured in 5% BCS/5% FBS α-MEM without phenol red (Gibco) on Collagen type 1a (MP Biomedicals) coated plastic plates10,16. HUVECs were purchased from ATCC and cultured in 5% Endothelial Growth Media (EGM, ScienCell), and human and murine multiple myeloma cell lines (MM1.S, JJN3, RPMI-8226, and 5TGM1) were cultured in 10% FCS RPMI (Gibco) with 1% Pen-strep. Ocys and MM cells were co-cultured at a 1:5 ratio as previously published^10^. Primary Human Endothelial Colony Formation Cells (ECFCs) were generously provided by Dr. Laura Haneline (IUSM). ECFCs were isolated from umbilical cord blood samples by the Indiana University Simon Cancer Center Angio BioCore and stored as previously described^66^. ECFC aliquots were thawed and cultured in endothelial growth media 2 (EGM2, Lonza America, Inc., Walkersille, MD) plus 10% FBS, plated on flasks coated with type 1 collagen (Corning, Inc., Durham, NC). Cells were passaged 2–5 times before being detached using 0.05% trypsin and resuspended at 2 × 10^5^ cells/mL EGM2. All cells were maintained at 37 °C with 5% CO_2_.

### Gene expression

mRNA levels were detected using SYBR Green technology. RNA was extracted from cells using TRIzol methodology and converted to cDNA using Applied Biosciences High Capacity cDNA Kit^67^. The ∆∆Ct method was used to calculate the fold change in gene expression relative to *Gapdh* and *Hprt1*. A list of primer sequences is provided in Supplemental Table S1*.* The Mouse Angiogenesis Gene qPCR Array was purchased from Qiagen (PAMM-024ZD-2) and used according to manufacturer’s instructions. Murine *Fgf23* expression was detected using Taqman technology relative to *Gapdh* levels (Mm02445621 and Mm99999915).

### Vegf-a knockdown

15,000 MLOA5 osteocytic cells were plated on collagen-coated 24 well plates and transfected with a Dharmacon siRNA ON-TARGET SMARTpool, a solution containing 4 specific siRNAs targeting Vegf-a, or with an ON-TARGET scramble sequence as per the manufacturer’s instructions (Horizon Discovery, Waterbeach, UK). Vegf-a knockdown was confirmed via mRNA expression, and siGapdh was used as a methodological positive control. siRNAs were used at 25 nM final concentration. Cell viability was assessed by Trypan Blue staining with a TC20 Automated Cell Counter (Bio-Rad).

### Hypoxic culture conditions

30,000 osteocytes (MLOA5s, MLOY4s, or primary calvariae) per well were cultured for 24 h in collagen-coated 24-well plates (MP Biomedicals, #160084) in an environment of 5% CO_2_ and 1% O_2_.

### Tube formation assays

96 well plates were coated with 50 µLs of growth-factor free basement membrane extract (Cultrex). After 30 min of incubation at 37 °C, 5000 ECFCs or HUVECs were plated on the coated wells in the presence of various conditioned media with/without anti-mVegf164 (R&D Systems, #AF493SP) or Normal Goat IgG (R&D Systems #AB108C) at 4.5 ng/mL. All conditioned media were diluted at a 1:1 ratio of 5% MLOA5 medium and 0% Endothelial medium. Networks were imaged with an EVOS XL microscope at 4 h, 8 h, and 24 h. 8 h images were analyzed for tube length and branching points.

### Transwell migration assays

Conditioned Media (200µLs) from MLOA5 cells cultured for 24 h in normoxia (21% O_2_) or hypoxia (1% O_2_) were added to individual wells of a 96-well plate. 5000 HUVECs were seeded onto the insert of a transwell and placed on top of the wells of the 96-well plate in 50µLs of 0% Endothelial Cell Medium. After 24 h, the transwell inserts were fixed with 10% formalin, washed, and stained with 0.2% Crystal Violet. Cells were counted and analyzed via EVOS XL microscopy at 4 × and 10 × magnifications.

### Western blots

Protein lysates were harvested using RIPA buffer. 30 µgs of protein were assessed for Vegf-a using a murine monoclonal antibody (Santa Cruz, Sc-53462, 1:200); β-actin and VCP were used as loading controls (SC-47778, abcam #11433, 1:1000). Precision Plus Protein Kaleidoscope molecular ladder from Bio-Rad was used for size analysis (Bio-Rad #161-0375). Membranes were imaged using a Bio-Rad ChemiDoc System and analyzed via Image Lab Software (Bio-Rad).

### Vegf-a ELISAs

Secreted Vegf-a levels were determined in conditioned media from mono-culture or co-cultures of MLOA5s and various MM cell lines using a Mouse Vegf-a ELISA according to manufacturer’s instructions (R&D Systems, #MMV00).

### In vivo myeloma studies

6-week old C57Bl/KalRij (Radl) female mice were injected intratibially with 100,000 syngeneic murine 5TGM1 myeloma cells/0.1 mL or saline. Tumor burden was monitored by assessing serum levels of monoclonal IgG2b, and bone lesions determined radiologically as previously described^10^. Mice were sacrificed at 10 weeks of age. For human myeloma cell experiments, 6-week old B.6CB17-Prkdscid/SzJ Scid female mice (Jackson Laboratories, Bar Harbor, Maine, USA) were injected intratibially with 100,000 human JJN3 myeloma cells/0.1 mL or saline then sacrificed 4 weeks later at 10-weeks of age^16^. Injected tibias were harvested for histological and immunohistochemical analyses. The Institutional Animal Care and Research Advisory Committee (IACUC) at IUSM approved all animal housing and experiments (#11448). All listed experiments were performed in accordance with the guidelines and regulations of IACUC at IUSM.

### Immunohistochemistry of murine tibiae

Paraffin-embedded tibiae from MM-bearing mice or controls were stained for murine Vegf-a (#SC-57426, 1:100), Endomucin-HRP (SC-65495, 1:500), or Cd31 (abcam 124432, 1:1000), and counterstained with hematoxylin. Vectorstain Labs kits (#PK-6102) was used to amplify the signal using a secondary antibody (#SC-2005). Sections were imaged on a Leica DM3000 upright microscope^21^.

### Fgf23 treatment

MLOA5 osteocyte-like cells were treated with 100 ng/mL of recombinant murine Fgf23 (Peprotech, #450-55) for 2, 6, and 24 h. Solid Fgf23 was diluted in MilliQ H_2_O at 0.5 mg/mL. Vehicle solution for working stock was PBS with 0.2% Bovine Serum Albumin. RNA and conditioned media were collected for gene expression and Vegf-a ELISA analyses.

### Primary Ocys and EVOCA assays of calvarial bones from Vegfa-^*flox/flox*^ and Fgf23 conditional knockout mice

Primary Ocys were isolated from *Vegfa-*^*flox/flox*^ adult mouse calvariae, using Sequential Collagenase P digestions^68,69^. Fractions 7–10 were pooled and plated as primary Ocys. After 48–72 h of culture, primary Ocys were passaged, and 15,000 cells were plated for co-culture experiments with different multiple myeloma cell lines for 48hrs. For ex vivo calvarial bone cultures (EVOCA), 5 mm biopsies of calvariae from *Vegfa-*^*flox/flox*^ mice were digested and plated in a 96 well plate as previously described^10^. Adeno-Cre-eGFP (Vegf-a knockdown) and Adeno-CMV-eGFP (Control) viruses were used at a multiplicity of infection of 120 for primary Ocys, and at a 7.8% solution for EVOCA^70^. 5 mm calvarial biopsies were also isolated from mice lacking Fgf23 specifically in osteocytes (*Fgf23 (∆/flox);Dmp1-Cre,* [Fgf23cKO]) for EVOCA studies and co-cultured with human JJN3 MM cells^28^. All EVOCA studies were performed using 50,000 MM cells^10,17^.

### Statistical analyses

Statistical analyses were performed using Graphpad Prism software (Irvine, CA, USA). All data are presented as means ± standard error. For experiments comparing two groups, we used a two-tailed unpaired t-test unless stated otherwise in the figure legends. For experiments comparing 3 or more groups, an ANOVA was used with a Tukey post-hoc test unless otherwise stated in the figure legend. A *p value* of less than 0.05 was considered significant, and the data are representative of at least 3 biological replicates.

## Supplementary information

Supplementary Information.
