# Supplementary material for: Osteocyte Vegf-a contributes to myeloma-associated angiogenesis and is regulated by Fgf23
Source: Sci Rep. 2020 Oct 14;10:17319. doi: 10.1038/s41598-020-74352-x (PMC7560700; doi:10.1038/s41598-020-74352-x)

*Osteocyte Vegf-a Contributes to Myeloma-associated Angiogenesis and Is Regulated by Fgf23*

Patrick L. Mulcrone, PhD^1, 4^, Shanique K. E. Edwards, PhD^5^, Daniela N. Petrusca, PhD^2^, Laura S. Haneline, M.D.^5^, Jesús Delgado-Calle, PhD^2, 3, 4^ and G. David Roodman, M.D., PhD^2, 4, 6, *^

1 Department of Microbiology and Immunology, 2 Department of Medicine, Division of Hematology/ Oncology, 3 Department of Anatomy, 4 Indiana Center for Musculoskeletal Health, 5 Department of Pediatrics, Indiana University School of Medicine, Indianapolis, IN, 6 Roudebush VA Medical Center, Indianapolis, IN, * Corresponding Author

**Corresponding Author**:

G. David Roodman, M.D, PhD

Joseph E. Walther Hall, Room C312
980 W. Walnut St.
Indianapolis, IN 46202

Phone: 317-274-3589

Fax: 317-274-0396

[groodman@iu.edu](mailto:groodman@iu.edu)


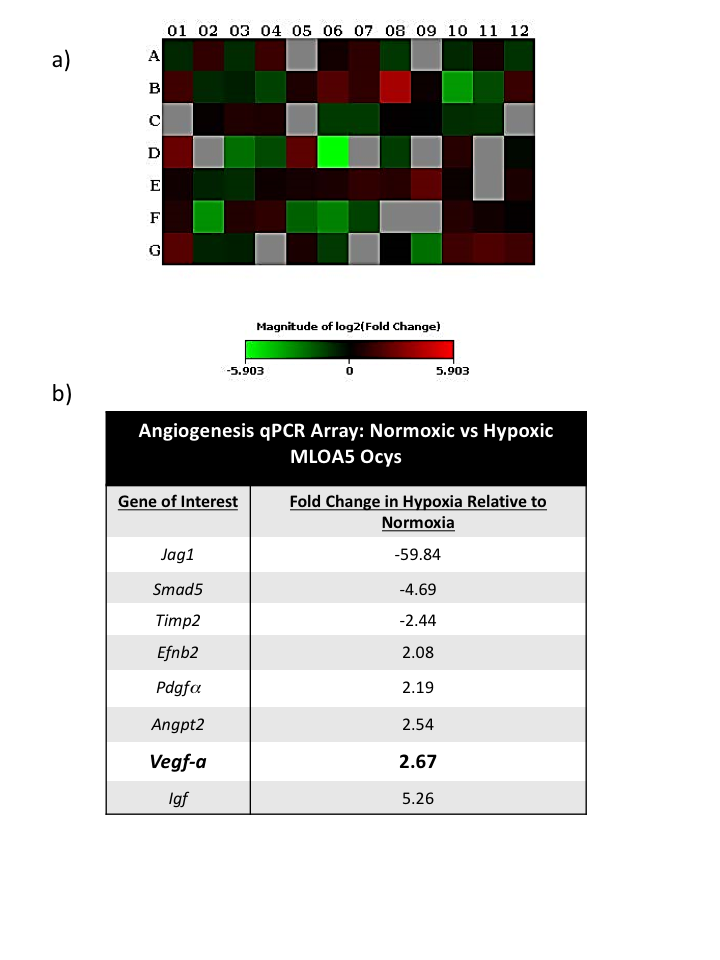


**Supplemental Figure S1. Osteocytes Increase expression of Pro-angiogenic Genes in Hypoxia.** a) Mouse Angiogenesis qPCR heat map of 84 genes comparing normoxic (21% O2) vs hypoxic (1% O2) culture conditions for MLOA5 osteocytes. Red indicates increased expression, while green shows reduced gene expression in hypoxia**.** *Gusb* was selected by software analysis as the housekeeping gene. b) Fold changes of selected genes of interest from hypoxic cultures at 24hrs are listed in the table from lowest to highest.


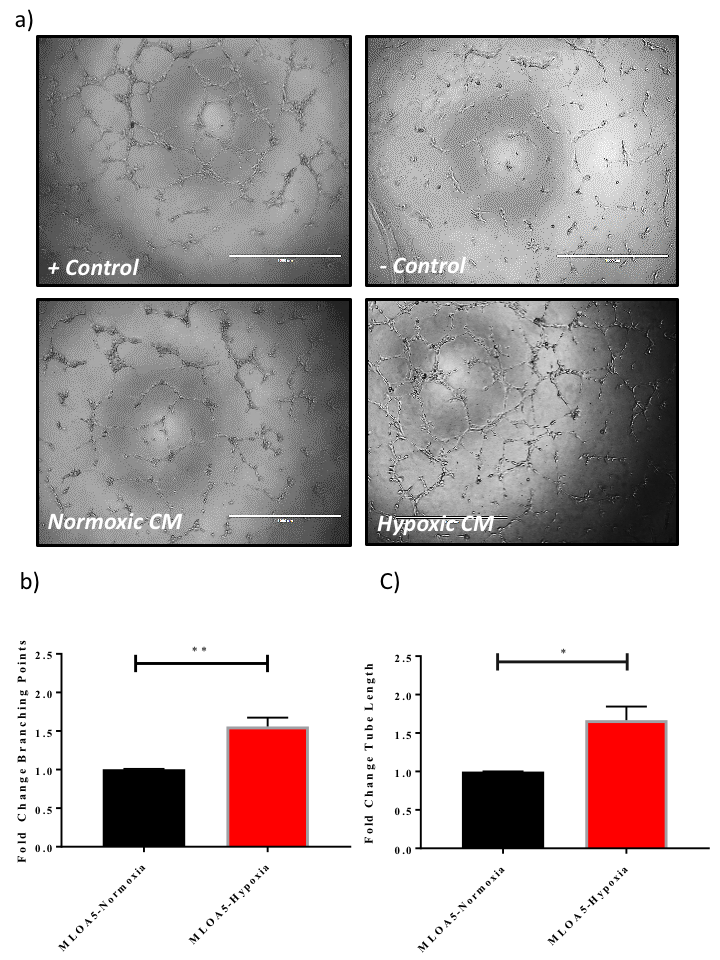


**Supplemental Figure S2**: **CM from MLOA5s Cultured in Hypoxia Promote HUVEC Tube Formation.** a) Representative 4x images of 5,000 HUVECs after 8hrs of treatment. b) Quantification of tube length and c) branching points of HUVECs (N=4). (* = *p*<0.05, **= p<0.01).


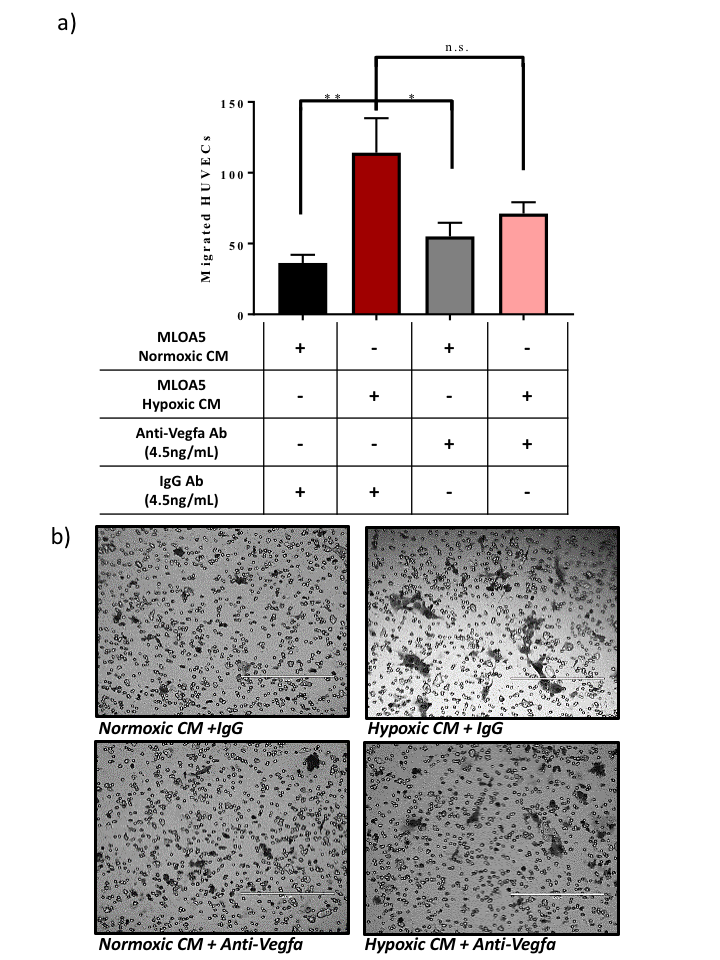


**Supplemental Figure S3**. **Hypoxic CM from Osteocytes Promotes HUVEC Transwell Migration.** a) Number of migrated HUVECs detected by crystal violet staining after 24hrs. Addition of the Vegf neutralizing antibody reduces migration number. Kruskal-Wallace P= 0.0063, (* = *p*<0.05, **= p<0.01). b) Representative 4x images (N=4-5)


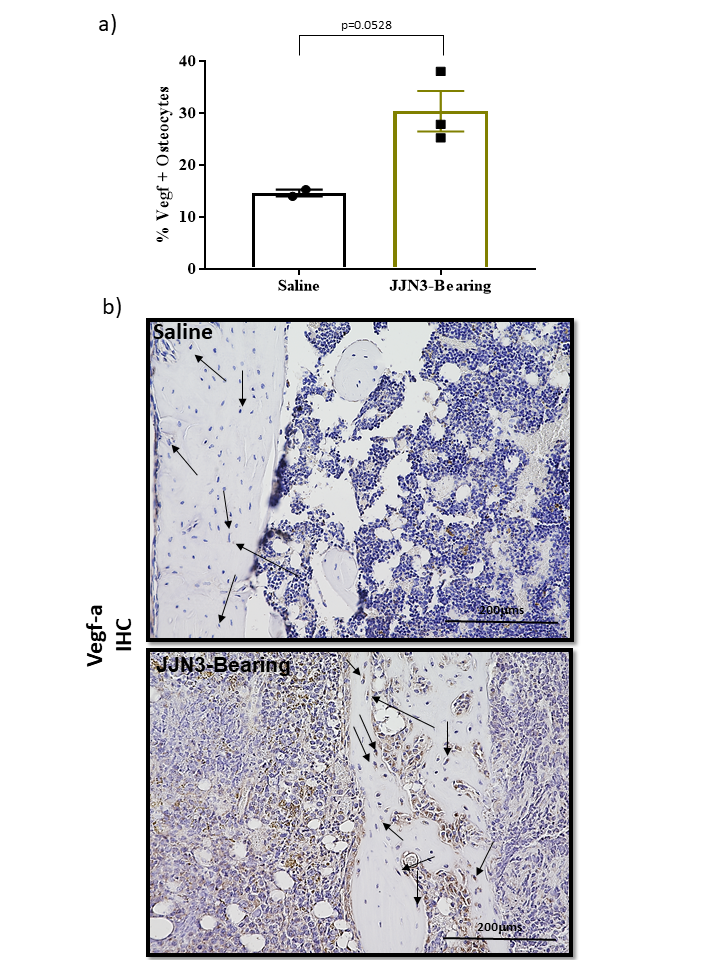


**Supplemental Figure S4.** **Bones Harboring JJN3 Cells Have Increased % of Vegf-a-positive Osteocytes**. a) Percentage of Vegf-positive osteocytes 4 weeks after inoculation. N=2 for control, 3 for tumor group. p=0.0528. b) Representative 20x images of Vegf-a-stained murine tibiae injected intratibially with saline or 10^5 JJN3 MM cells. Black arrows indicate Vegf-a-positive osteocytes.


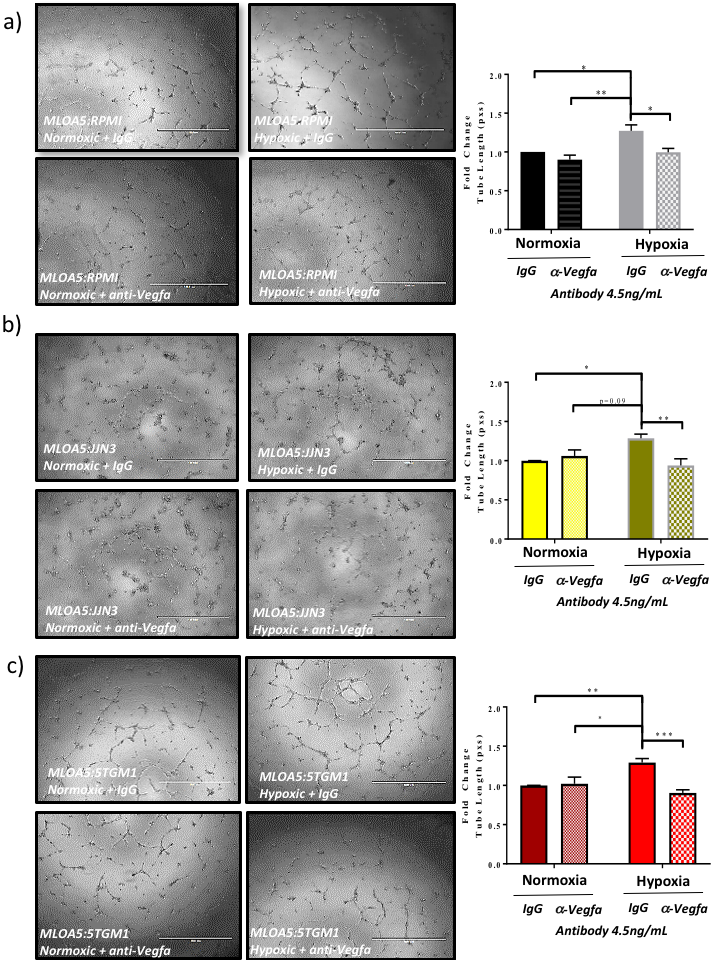


**Supplemental Figure S5. Conditioned Media from Hypoxic MM:MLOA5 Co-cultures Induce Vessel Formation at 8hrs.** Increased HUVEC vessel length measured with co-culture conditioned media between MLOA5s & RPMIs (a, N=3), JJN3s (b, N=5), or 5TGM1s (c, N=5). Addition of murine Vegf-a neutralizing antibody blunts pro-angiogenic effects of Hypoxic CM in all conditions. a) 4x images of MLOA5:RPMI co-cultures with graphed fold change tube length. b) 4x images of MLOA5:JJN3 co-cultures with graphed fold change tube length. c) 4x Images of MLOA5:5TGM1 co-cultures with graphed fold change tube length. (* = *p*<0.05, **= p<0.01, ***= p<0.001 by ANOVA).


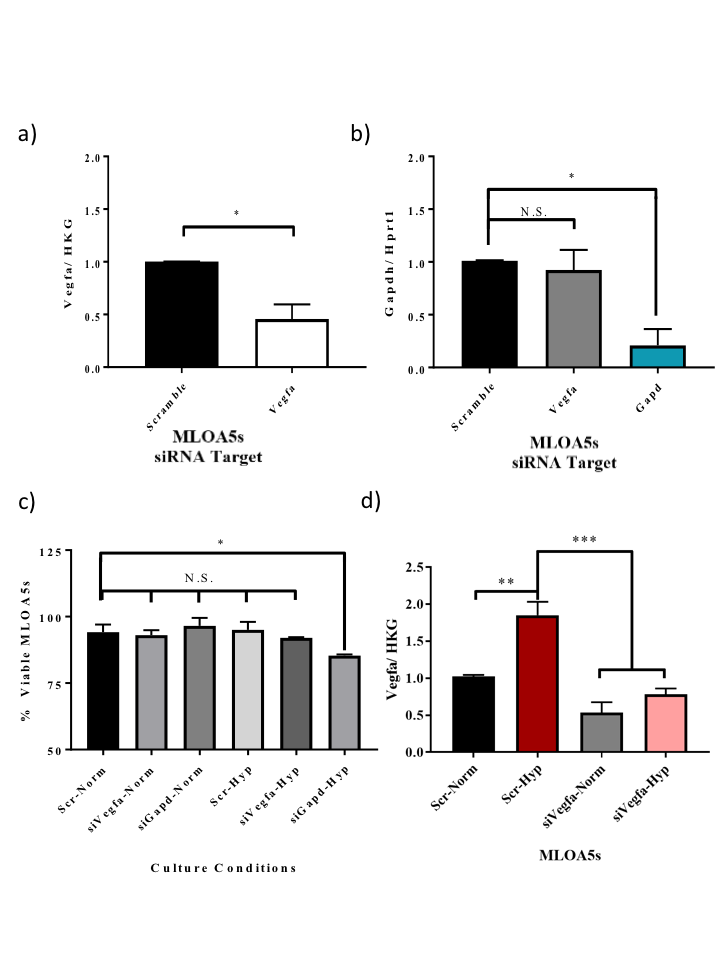


**Supplemental Figure S6. Knockdown Efficiency of Vegf-a in and Viability of MLOA5 Osteocytes.** a) siRNA targeting Vegf-a results in ~55% reduction in mRNA levels b) Targeting Gapdh has no effect of Vegf-a expression in MLOA5s. c) Trypan Blue counts for different siRNA groups. (* = *p*<0.05). d) scr-MLOA5s increase Vegf-a expression in hypoxia, while siVegfa-MLOA5s do not. A baseline decrease in Vegf-a expression is observed in normoxic siVegfa-MLOA5s. N=3 for each experiment. (**= p<0.01, ***= p<0.001 by ANOVA for Panel d).


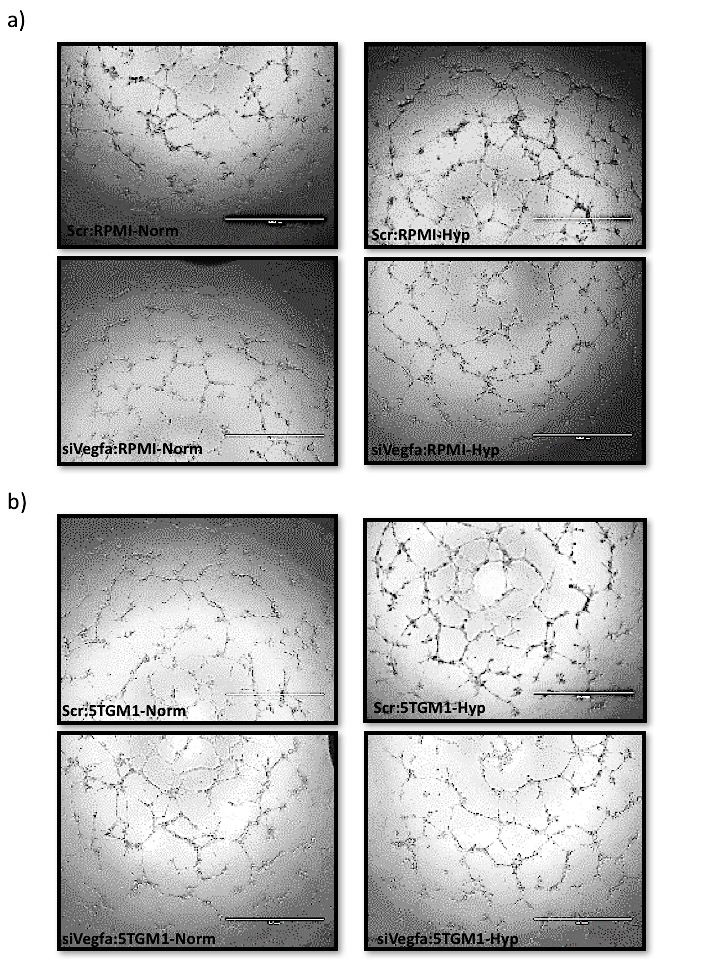


**Supplemental Figure S7. Representative 8hr Images of HUVECs treated with various siRNA-MLOA5:MM Co-Culture CM.** a) Representative images of HUVECs treated with RPMI:MLOA5 CM with either Scr or siVegfa MLOA5s, 4x magnification. b) Representative images of HUVECs treated with 5TGM1:MLOA5 CM with either Scr or siVegfa MLOA5s, 4x magnification.


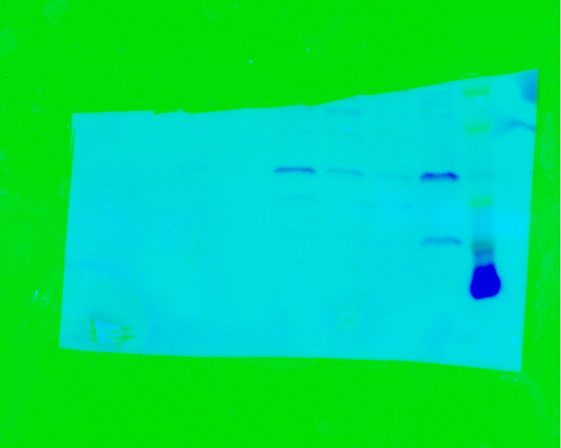

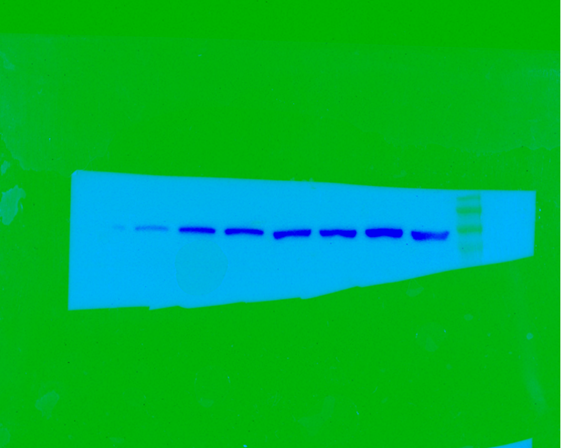


10

9

8

7

6

5

4

3

2

1

Lane:

*Vcp*

100kDa

*Vegf-a*

50kDa

37kDa

20kDa

10kDa

**Supplemental Figure S8. Original Western Blot Images Re: Figure 1c.** The gel was laid in reverse for the transfer step. Therefore, the image was rotated for final display in Figure 1c. Lane 1 is empty. Lane 2 is 15µLs of the molecular ladder (Bio-Rad #161-0375). Lane 3 is the U266 cell line, which was run as a positive control for Vegf-a. Lane 4 is *MLOA5-Normoxia* sample analyzed for Figure 1c. Lane 5 is *MLOA5-Hypoxia1* sample analyzed for Figure 1c. Lane 6 is *MLOA5-Hypoxia2* sample analyzed for Figure 1c. Lanes 7-10 are the MM1.S cell line, used for a different study, and were not relevant or analyzed for this manuscript submission. Exposure time for Vcp is 45s, and size is predicted to be 100kDa according to manufacturer. Exposure time for Vegf-a is 120s, and sizes are predicted to be 42kDa for the dimer and 21kDa for the monomer according to manufacturer. Blue arrows indicate Vegf-a monomer and dimer bands. Ladder sizes indicated to the right of Lane 1. This blot is representative of 2 experimental blots.


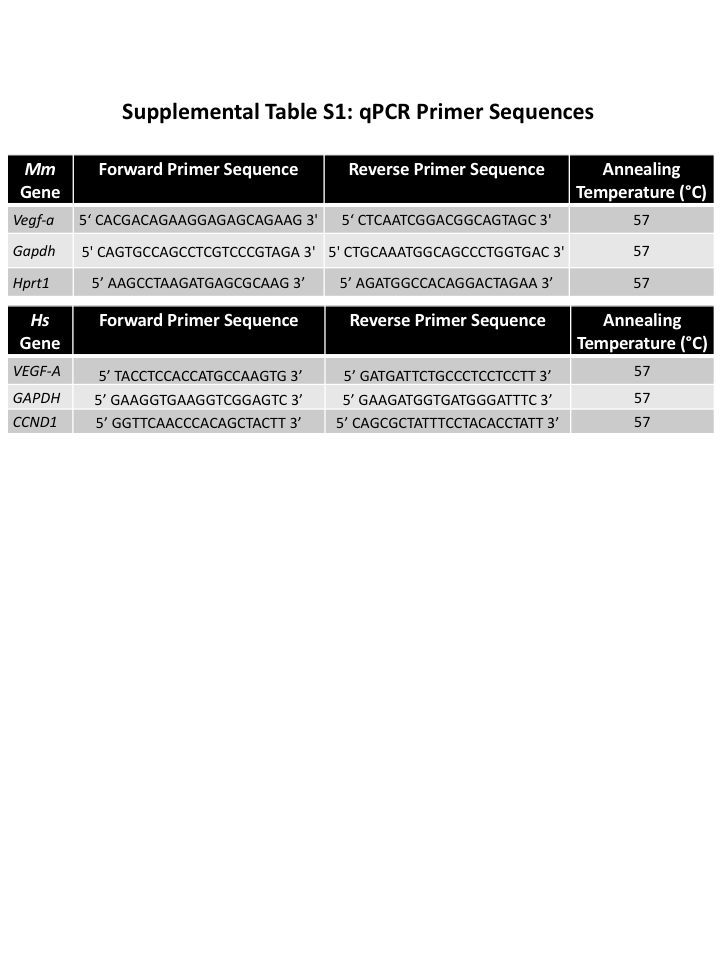

Supplement: Supplementary file 1 — Supplementary Information. [file 41598_2020_74352_MOESM1_ESM.docx]
